# Supplementary material for: Mulberry Leaf Extract Improves Metabolic Syndrome by Alleviating Lipid Accumulation In Vitro and In Vivo
Source: Molecules. 2022 Aug 11;27(16):5111. doi: 10.3390/molecules27165111 (PMC9416217; doi:10.3390/molecules27165111)
Supplement: Supplementary file 1 [file molecules-27-05111-s001.zip › molecules-1841242-supplementary.pdf]

Supporting Information

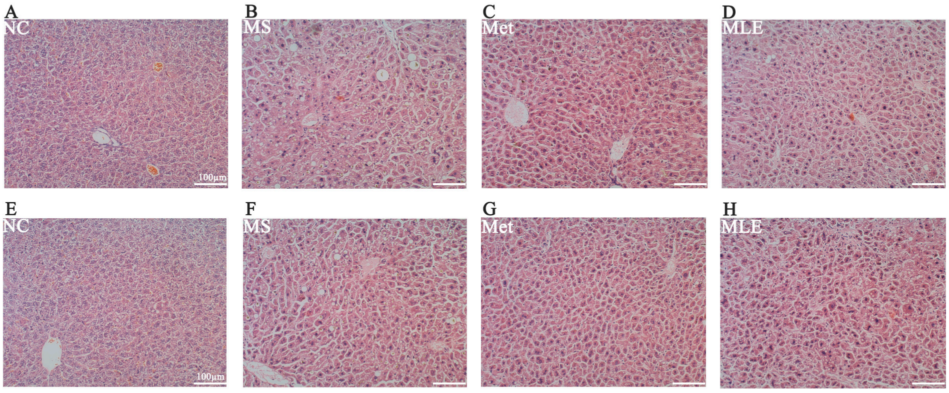

Figure S1. The effect of MLE on the liver tissue of mice with MS. The mice in the control group were administered with 0.9% NaCl (NC), while MS mice were administered with 0.9% NaCl (MS), metformin (200 mg/kg/d, Met), and MLE (100 mg/kg/d, MLE) by oral gavage for 15 weeks. After the intervention, the liver tissues of each group of mice were collected, fixed in 4% paraformaldehyde, and stained with hematoxylin-eosin. HE staining of liver tissues of different individuals in the NC group (A,E), MS group (B,F), Met group (C,G) and MLE group (D,H). Scale bar: 100 μM.

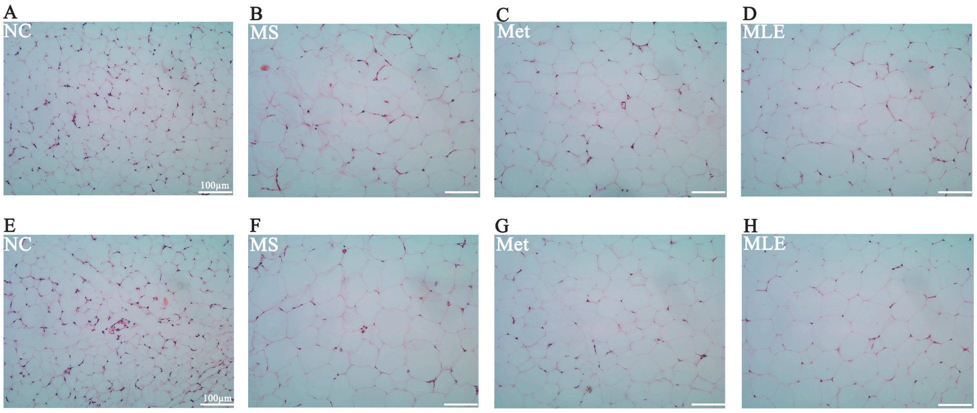

Figure S2. The effect of MLE on the adipose tissue of mice with MS. The mice in the control group were administered with 0.9% NaCl (NC), while MS mice were administered with 0.9% NaCl (MS), metformin (200 mg/kg/d, Met), and MLE (100 mg/kg/d, MLE) by oral gavage for 15 weeks. After the intervention, the adipose tissues of each group of mice were collected, fixed in 4% paraformaldehyde, and stained with hematoxylin-eosin. HE staining of epididymal adipose tissue of different individuals in the NC group (A, E), MS group (B, F), Met group (C, G) and MLE group (D, H). Scale bar: 100 μM.
